# Optimized RTX strategy plus structured glucocorticoid tapering for primary membranous nephropathy: a multicenter propensity score-matched cohort study

**DOI:** 10.3389/fmolb.2026.1770916

**Published:** 2026-03-04

**Authors:** Yao Sun, Yuxia Zhang, Jing Liu, Yanting Yu, Min Wu, Qing Yin, Yujia Wang, Ziyu Liang, Biao Huang, Ri-Ning Tang, Hai-ming Xia

**Affiliations:** 1 School of Medicine, Southeast University, Nanjing, China; 2 Department of Nephrology, Nanjing Drum Tower Hospital, Medical School of Nanjing University, Nanjing, China; 3 College of Life Sciences and Medicine, Zhejiang Sci-Tech University, Hangzhou, China; 4 Nanjing Drum Tower Hospital, Medical School of Nanjing University, Nanjing, China

**Keywords:** anti-PLA2R antibodies, glucocorticoid tapering, pharmacokinetics, primary membranous nephropathy, rituximab, therapeutic drug monitoring

## Abstract

**Background:**

Standard rituximab (RTX) regimens for primary membranous nephropathy (PMN) may result in subtherapeutic RTX exposure within 2–3 months due to altered pharmacokinetics, potentially contributing to delayed remission, incomplete immunologic control, and relapse. We evaluated whether an exposure-optimized RTX strategy combined with structured glucocorticoid tapering was associated with improved clinical and immunologic outcomes in PMN.

**Methods:**

This multicenter retrospective study included 182 PMN patients with nephrotic syndrome (2020–2025). After 1:2 propensity score matching, 75 patients were analyzed: an exposure-optimized strategy group (RTX 375 mg/m^2^ on days 1, 15, 30, and 120 with structured prednisone tapering, with subsequent TDM-guided redosing when RTX <2 μg/mL) versus standard RTX (RTX 375 mg/m^2^ weekly ×4 weeks). Median follow-up time was 17.0 (IQR: 12.5–25.6) and 14.8 (IQR: 12.0–27.1) months for GC/MRTX and SRTX groups, respectively. Primary endpoint: complete remission (CR; proteinuria <0.3 g/24 h). Secondary endpoints: near-CR (NCR; ≥80% proteinuria reduction), complete immunological remission (anti-PLA2R < 2 RU/mL), and relapse.

**Results:**

At 6 months, the GC/MRTX group had higher RTX concentrations (median 7.46 vs. 0.07 μg/mL, p = 0.020) and a higher proportion of patients with RTX concentrations ≥2 μg/mL (60.0% vs. 21.1%, p = 0.022). Anti-RTX antibodies were detected only in the SRTX group (11%). At 12 months, GC/MRTX was associated with higher CR (64.0% vs. 22.0%, p < 0.001), higher complete immunological remission (80% vs. 42%, p = 0.002), and shorter time to CR (9.0 vs. 18.4 months, p < 0.001). NCR at 12 months was 88.0% versus 70.0% (p = 0.085). Over follow-up, GC/MRTX showed higher cumulative CR (p < 0.001) and NCR (p = 0.036) and lower relapse (0% vs. 18.4%, p = 0.026). In refractory PMN (n = 51), GC/MRTX achieved higher 12-month CR (52.63% vs. 18.75%, p = 0.012) and complete immunological remission (89.47% vs. 34.38%, p = 0.001). Safety profiles were comparable.

**Conclusion:**

In this propensity score–matched multicenter cohort, an exposure-optimized strategy combining interval RTX dosing, structured glucocorticoid tapering, and TDM-guided redosing was associated with higher and earlier remission, deeper immunologic response, and lower relapse compared with the standard RTX monotherapy.

## 1 Introduction

Primary membranous nephropathy (PMN), characterized by diffuse thickening of the glomerular basement membrane and subepithelial immune deposits, is a leading cause of nephrotic syndrome in adults (Couser, 2017; Ponticelli and Glassock, 2014). With the discovery of the M-type phospholipase A2 receptor (PLA2R) as the principal autoantigen (Beck et al., 2009), PMN is currently established as an autoimmune disease driven by pathogenic autoantibodies and B-cell dysregulation (van de Logt et al., 2019). Rituximab (RTX), an anti-CD20 monoclonal antibody, depletes B cells and reduces autoantibody production, with its efficacy confirmed in randomized controlled trials (Fervenza et al., 2019; Scolari et al., 2021; Fernández-Juárez et al., 2021). The MENTOR trial demonstrated the superiority of RTX over cyclosporine in achieving sustained remission (Fervenza et al., 2019), leading to its endorsement as first-line therapy in the KDIGO 2021 guidelines (Kidney Disease: Improving Global Outcomes KDIGO Glomerular Diseases Work Group, 2021). Despite this, systematic reviews and real-world studies have revealed suboptimal complete remission (CR) rates with standard RTX (SRTX) monotherapy in PMN patients with nephrotic syndrome (Bomback et al., 2009; Ruggenenti et al., 2012; Huang et al., 2025), suggesting significant room for optimization of dosing regimens and treatment intervals.

A growing body of real-world pharmacokinetic evidence provides insight into these suboptimal outcomes. Several studies have demonstrated that patients with PMN—particularly those with nephrotic-range proteinuria—experience markedly altered RTX pharmacokinetics, including accelerated clearance, shortened half-life, and early decline of serum RTX concentrations to subtherapeutic levels (<2 μg/mL) within 2–3 months after standard weekly ×4 infusions (Dahan et al., 2017; Fogueri et al., 2019; Boyer-Suavet et al., 2019). These abnormalities have been attributed to non-selective urinary immunoglobulin loss associated with heavy proteinuria, altered distribution, and high immunologic target burden. The development of anti-RTX antibodies (ARAs) further accelerates RTX elimination, impairs immunologic control, and increases relapse risk, particularly in patients with low drug exposure (Boyer-Suavet et al., 2019). Collectively, these findings highlight a consistent “pharmacokinetic–immunologic vulnerability window” at approximately 2–3 months post-infusion, during which RTX levels fall below therapeutic thresholds while B-cell reconstitution and PLA2R antibody rebound may occur (Dahan et al., 2017; Fogueri et al., 2019; Boyer-Suavet et al., 2019).

Recent model-informed analyses support exposure-oriented individualization in autoimmune glomerular diseases. In a population pharmacokinetic model, body weight, proteinuria, disease type, treatment duration, and anti-RTX antibody formation were key determinants of RTX exposure; accordingly, dosing schedules (including interval adjustment in high-proteinuria, difficult-to-treat diagnoses such as PMN) were recommended to improve exposure reliability (Hartinger et al., 2024). Real-world therapeutic drug monitoring (TDM) data from regional laboratories in China further show frequent subtherapeutic RTX exposure and anti-RTX antibody formation in PMN (Supplementary Figure 1). Together, these findings highlight the limitations of fixed-interval standard RTX dosing in PMN and support optimized regimens to maintain adequate exposure throughout the vulnerability period (Dahan et al., 2017; Fogueri et al., 2019; Boyer-Suavet et al., 2019; Hartinger et al., 2024).

Another limitation of SRTX monotherapy is delayed clinical response. Whereas B-cell depletion after RTX requires several weeks to translate into clinical improvement, glucocorticoids provide rapid control of proteinuria. Although KDIGO guidelines emphasize minimizing long-term glucocorticoid exposure (Kidney Disease: Improving Global Outcomes KDIGO Glomerular Diseases Work Group, 2021), recent studies demonstrate that structured low-dose glucocorticoid tapering regimens can maintain disease control with improved safety profiles in nephrotic syndrome (Li et al., 2024). The combination of RTX with structured glucocorticoid tapering may therefore yield synergistic effects—providing early disease control while potentially suppressing ARA formation—and limiting steroid-related adverse events.

Informed by these observations, we developed a modified RTX (GC/MRTX) plus glucocorticoid tapering regimen: RTX 375 mg/m^2^ administered on days 1, 15, 30, and 120, with concomitant prednisone initiated at 0.5 mg/kg/day, tapered to 20 mg/day by month 3, gradually reduced to 5 mg/day by month 6, and maintained thereafter. Following this initial course, serum RTX concentrations were monitored every 3 months, with additional infusions administered when drug levels fell below the therapeutic threshold of 2 μg/mL. This pharmacokinetic-guided strategy aims to maintain adequate drug exposure throughout the vulnerability period, prevent premature B-cell reconstitution, and suppress ARA formation (Dahan et al., 2017; Fogueri et al., 2019; Boyer-Suavet et al., 2019; Hartinger et al., 2024). In this study, we compared the efficacy and safety of GC/MRTX versus SRTX in PMN patients with nephrotic syndrome, specifically evaluating clinical remission, immunological remission, relapse rates, RTX pharmacokinetics, and ARA formation.

## 2 Materials and methods

### 2.1 Study population

**FIGURE 1:**
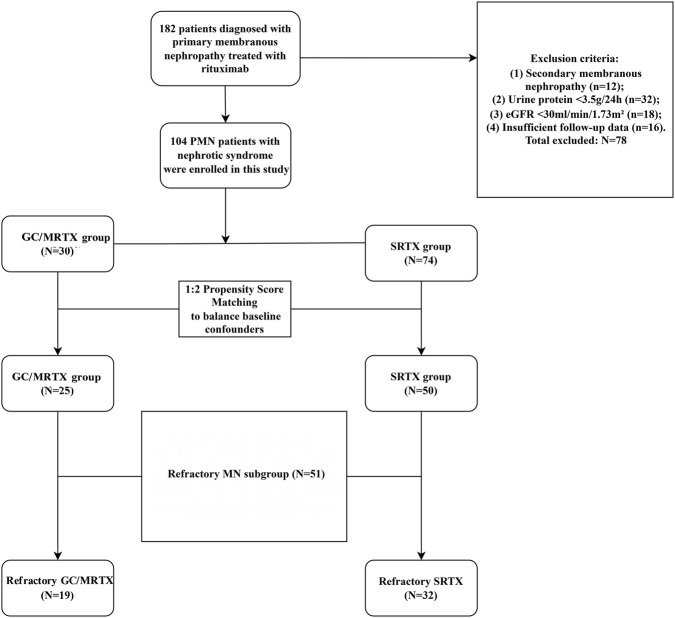
Study flowchart of patient selection and matching. This flow diagram shows the screening strategy, inclusion and exclusion criteria, and the 1:2 propensity-score matching procedure. A total of 75 matched primary membranous nephropathy (PMN) patients were included (GC/MRTX, n = 25; SRTX, n = 50). A predefined refractory membranous nephropathy (RMN) subgroup (n = 51) was additionally identified.

This multicenter retrospective study included patients with PMN who were treated between September 2020 and September 2025 at the Department of Nephrology of Zhongda Hospital, affiliated with Southeast University, and the Department of Nephrology of Nanjing Drum Tower Hospital, affiliated with the Medical School of Nanjing University. Of the 182 patients screened, 104 fulfilled the following inclusion criteria (Figure 1): (1) renal biopsy showing PMN, or aPLA2Rab ≥14 RU/mL; (2) age between 18 and 80 years; (3) nephrotic syndrome at baseline, defined as persistent nephrotic-range proteinuria (24-h urinary protein ≥3.5 g) with serum albumin <30 g/L; (4) receiving either the GC/MRTX or SRTX treatment regimen with completed follow-up; (5) follow-up time ≥12 months. The exclusion criteria were as follows: (1) secondary membranous nephropathy caused by connective tissue disease, malignancy, or hepatitis B; (2) refusal of follow-up. Definition of refractory PMN (RMN subgroup). The prespecified RMN subgroup comprised patients with treatment-resistant and/or relapsing PMN, defined as failure to achieve at least partial remission after an adequate prior course of immunosuppressive therapy, and/or relapse after a documented remission.

This study was approved by the Ethics Committee of Zhongda Hospital, Southeast University (Approval No. 2025ZDSYLL189-P01), and was conducted in accordance with the Declaration of Helsinki (1964 and its later amendments). Given the retrospective nature of the study and the use of de-identified clinical data, the requirement for written informed consent was formally waived by the Ethics Committee of Zhongda Hospital, Southeast University. The ethics committee confirmed that the waiver met national and institutional guidelines and complied with the Declaration of Helsinki.

### 2.2 Treatment regimens

**FIGURE 2:**
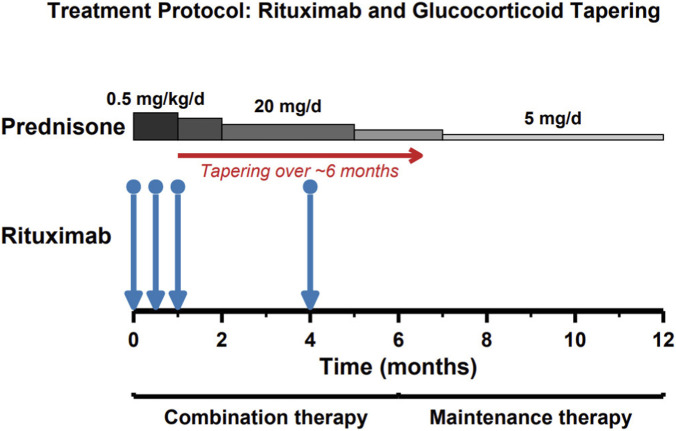
Treatment regimens for the modified-interval rituximab (GC/MRTX). The GC/MRTX regimen consisted of rituximab (RTX) 375 mg/m^2^ administered on days 1, 15, 30, and 120, combined with structured prednisone tapering to 5 mg/day by month 6.

The exposure-optimized strategy (GC/MRTX) is illustrated in Figure 2 and consisted of RTX 375 mg/m^2^ administered intravenously on days 1, 15, 30, and 120. After completion of the induction course, serum rituximab concentrations were measured every 3 months, and TDM-guided redosing was performed as a single additional infusion of RTX 375 mg/m^2^ when the serum RTX concentration fell below 2 μg/mL. Because a clinically validated TRFIA target concentration has not been established for PMN, this cutoff was used to standardize real-world redosing decisions rather than to represent an evidence-based therapeutic target. Oral prednisone was initiated at 0.5 mg/kg/day, tapered to 20 mg/day by month 3, gradually reduced to 5 mg/day by month 6, and maintained thereafter. The SRTX monotherapy regimen comprised the administration of RTX 375 mg/m^2^ intravenously once weekly for four consecutive weeks without concomitant glucocorticoid therapy. Supportive measures, including renin–angiotensin system inhibitors, anticoagulants, lipid-lowering agents, and infection prophylaxis, were provided as indicated. Retreatment at month 6 consisted of RTX 375 mg/m^2^ per infusion. For partial responders, a single infusion was generally used for consolidation following shared decision-making (including patient preference and treatment affordability). For non-responders, a second infusion 1 week later (weekly ×2) could be administered at the treating physician’s discretion. Owing to the outbreak of the coronavirus disease 2019 in China and the hospitalization-control policies of medical insurance, as well as other factors, including the occurrence of infections and patients’ unwillingness to attend hospital visits, some patients were unable to receive RTX at scheduled timepoints. Consequently, the interval between RTX infusions in some patients with nephrotic syndrome was occasionally brought forward or delayed. For each planned rituximab administration timepoint (GC/MRTX: days 1, 15, 30, and 120; SRTX: weekly ×4), we recorded the planned date and the actual administration date. Timing deviation was defined as *(Actual date − Planned date)* in days; any non-zero value was considered a deviation (negative = earlier, positive = delayed). Deviations were summarized at both the administration level (by timepoint) and the patient level (any deviation, any earlier, any delay, and maximum absolute deviation across timepoints). Detailed tabulations are provided in the Supplementary Table 1.

### 2.3 Follow-up and data collection

Clinical data and laboratory indicators were collected at baseline and during standardized follow-up visits at 1, 2, 3, 6, 9, and 12 months. After 12 months, patients were followed at variable intervals based on clinical status. Median follow-up was 17.0 months (interquartile range [IQR]: 12.5–25.6) in the GC/MRTX group and 14.8 months (IQR: 12.0–27.1) in the SRTX group. For patients who could not attend scheduled hospital visits, information regarding remission status (based on patient-reported outcomes and available local laboratory reports) and relapse was obtained through structured telephone interviews. The CKD-EPI 2009 formula was used to assess the estimated glomerular filtration rate (eGFR). Peripheral blood B lymphocytes were detected using flow cytometry. B-cell depletion was diagnosed based on a CD19^+^ cell count <5 cells/μL. A quantitative enzyme-linked immunosorbent assay was used to detect aPLA2Rab concentrations, with immunological remission defined as aPLA2Rab levels <14 RU/mL and complete immunological remission as aPLA2Rab <2 RU/mL. For each nominal follow-up time point, the closest available CD19^+^ measurement within a ±4-week visit window was used. CR was defined as 24 h urinary protein <0.3 g, serum albumin ≥30 g/L, and an eGFR reduction of <25% compared with baseline. Near-CR (NCR) was defined as a proteinuria reduction of >80% compared with baseline. Partial remission (PR) was defined as proteinuria quantification that decreased by >50% compared with baseline, 24-hour urinary protein excretion <3.5 g, serum albumin concentration ≥30 g/L, and eGFR reduction of <25% compared with baseline. If the above indicators were not met, patients were classified as having no remission. The total remission rate (TR) includes CR or PR. Disease relapse was defined as the recurrence of nephrotic-range proteinuria (≥3.5 g/24 h) after achieving PR or CR. End-stage renal disease was diagnosed based on eGFR <15 mL/min/1.73 m^2^ with uremia, hyperkalemia, or implementation of renal replacement therapy. Severe infection was defined as an infection requiring >24 h hospitalization, requiring intravenous antibiotic therapy, or resulting in death.

### 2.4 Statistical analysis

Data are expressed as mean ± SD, median (IQR), or frequencies (percentages). Group comparisons were performed using Student’s t-test, Mann–Whitney U test, chi-square test, or Fisher’s exact test, as appropriate. Longitudinal laboratory parameters were summarized at each scheduled visit. Between-group comparisons at each visit were performed using Student’s t-test or Mann–Whitney U test, as appropriate, based on available cases at that time point (no imputation). Kaplan–Meier curves with log-rank tests compared time-to-event outcomes between groups. Cox proportional hazards regression identified independent predictors of CR. Propensity score matching (1:2, caliper 0.2) balanced baseline characteristics. Matching variables included age, sex, 24-h urinary protein, serum albumin, serum creatinine, eGFR, CD19^+^ B-cell count. Analyses were performed using R 4.4.2. P < 0.05 was considered significant. Balance metrics are provided in Supplementary Table 2.

## 3 Results

### 3.1 Baseline characteristics of the entire cohort

Baseline characteristics were well balanced after matching (Figure 1; Table 1). The median age was 59.0 years (IQR 50.0–67.0) versus 55.5 years (IQR 46.3–66.5) (p = 0.525). 24-h proteinuria was 10.8 g/24 h (IQR 6.4–13.6) versus 10.1 g/24 h (IQR 6.0–13.9) (p = 0.783). Serum albumin was 26.6 g/L (IQR 23.4–28.0) versus 24.6 g/L (IQR 22.3–27.9) (p = 0.325). eGFR was 86.4 mL/min/1.73 m^2^ (IQR 56.7–102.6) versus 90.6 mL/min/1.73 m^2^ (IQR 71.2–102.6) (p = 0.455), and anti-PLA2R positivity was 70.8% versus 74.0% (p = 0.774). No significant differences were observed in key clinical parameters.

**TABLE 1:** Baseline clinical characteristics.

	Before PSM			After PSM		
Variables	GC/MRTX	SRTX	P Value	GC/MRTX	SRTX	P Value
N	30	74		25	50	
Demographic						
Age (y)	58.0 (50.0, 66.5)	56.50 (42.3, 68.5)	0.664	59.0 (50.0, 67.0)	55.5 (46.3, 66.5)	0.525
Male (%)	19 (63.3)	56 (75.7)	0.204	17 (68.0)	34 (68.0)	1.000
BMI (kg/m^2^)	24.6 (22.1, 26.2)	24.2 (21.9, 26.6)	0.774	24.6 (22.0, 26.3)	24.2 (21.7, 26.9)	0.740
Medical history						
Hypertension (%)	20 (66.7)	48 (64.9)	0.861	15 (60.0)	31 (62.0)	0.867
Diabetes mellitus (%)	7 (23.3)	15 (20.3)	0.729	7 (28.0)	10 (20.0)	0.435
Cardiovascular Disease (%)	1 (3.3)	5 (6.8)	0.830	1 (4.0)	2 (4.0)	1.000
Cerebrovascular Disease (%)	1 (3.3)	11 (14.9)	0.184	1 (4.0)	8 (16.0)	0.258
Clinical variables						
Urine protein (g/24 h)	11.3 (6.6, 13.7)	9.3 (6.0, 13.8)	0.331	10.8 (6.4, 13.6)	10.1 (6.0, 13.9)	0.783
Albumin (g/L)	27.0 (23.4, 29.6)	23.9 (21.0, 27.9)	0.042*	26.6 (23.4, 28.0)	24.6 (22.3, 27.9)	0.325
Scr (μmol/L)	76.5 (65.8, 101.0)	83.2 (60.0, 112.8)	0.911	77.0 (70.0, 111.0)	79.5 (60.0, 96.5)	0.345
eGFR (mL/min/1.73 m^2^)	87.9 (57.0, 102.5)	93.5 (63.2, 106.5)	0.338	86.4 (56.7, 102.6)	90.6 (71.2, 102.6)	0.455
TG (mmol/L)	1.7 (1.2, 2.8)	1.9 (1.6, 2.8)	0.135	1.6 (1.1, 2.0)	2.0 (1.6,2.8)	0.054
TC (mmol/L)	6.6 (5.5, 7.7)	6.6 (5.4, 8.0)	0.857	7.0 (6.3, 8.2)	6.8 (5.7,8.8)	0.796
HDL (mmol/L)	1.5 (1.2, 1.9)	1.3 (1.0, 1.6)	0.015*	1.6 (1.3, 2.0)	1.4 (1.0,1.6)	0.063
LDL (mmol/L)	3.6 (3.1, 4.2)	3.6 (2.8, 4.7)	0.938	3.7 (2.8, 4.5)	3.7 (3.1,4.6)	0.580
CD3^+^ T cell (cell/μL)	998.9 (690.9, 1,527.2)	1,387.0 (951.4, 1778.1)	0.032*	1,222.44 (754.25, 1,554.67)	1,397.50 (959.30, 1790.46)	0.126
CD4^+^ T cell (cell/μL)	630.5 (453.0, 1,031.0)	839.0 (593.5, 1,139.0)	0.184	330.69 (211.25, 630.34)	910.76 (624.00, 1,128.48)	0.282
CD8^+^ T cell (cell/μL)	320.9 (216.4, 516.5)	480.5 (332.0, 639.2)	0.031*	275.42 (169.68, 331.58)	482.00 (341.72, 639.23)	0.104
CD19^+^ B cell (cell/μL)	274.1 (176.1, 329.4)	239.1 (169.3, 345.5)	0.963	275.42 (169.68, 331.58)	248.75 (155.50, 327.11)	0.822
PLA2R ab* (RU/mL)	36.2 (13.3, 69.9)	52.9 (21.9, 129.9)	0.189	36.1 (12.9, 54.5)	46.6 (13.2, 107.5)	0.240
PLA2R ab positive (%)	15 (50.0)	44 (59.5)	0.378	17 (70.8)	37 (74.0)	0.774

IQR represents interquartile range; PLA2R, phospholipase A2 receptor; eGFR, estimated glomerular filtration rate; GC/MRTX, exposure-optimized strategy consisting of modified-interval rituximab plus structured glucocorticoid tapering and subsequent TDM-guided redosing; SRTX: standard rituximab monotherapy regimen (RTX, 375 mg/m^2^ weekly ×4). *P < 0.05 indicates statistically significant differences between groups.

#### 3.1.1 Timing deviations from the planned infusion schedule

Deviations between planned and actual administration dates were observed in both groups and were predominantly delays. In the GC/MRTX cohort (n = 25), deviations increased at later infusions (Infusion 2–4: 64%, 76%, and 92% of administrations with any deviation, respectively), with delays being more common than earlier administrations. At the patient level, 23/25 (92%) had ≥1 deviation, 22/25 (88%) had ≥1 delay, and 6/25 (24%) had ≥1 earlier-than-planned administration; 5/25 (20%) experienced both earlier and delayed administrations across visits. In the SRTX cohort (n = 50), no deviations occurred at Week 1, whereas deviations were observed at Weeks 2–4 (40%, 58%, and 58%); all non-zero deviations were delays with no earlier administrations. The median patient-level maximum absolute deviation was 6 days (IQR 2–8; range 0–15) in GC/MRTX and 1 day (IQR 0–2; range 0–5) in SRTX. Detailed results by timepoint are shown in Supplementary Table 1A, and patient-level summaries are provided in Supplementary Table 1B.

### 3.2 Efficacy comparison between two treatments in PMN

**FIGURE 3:**
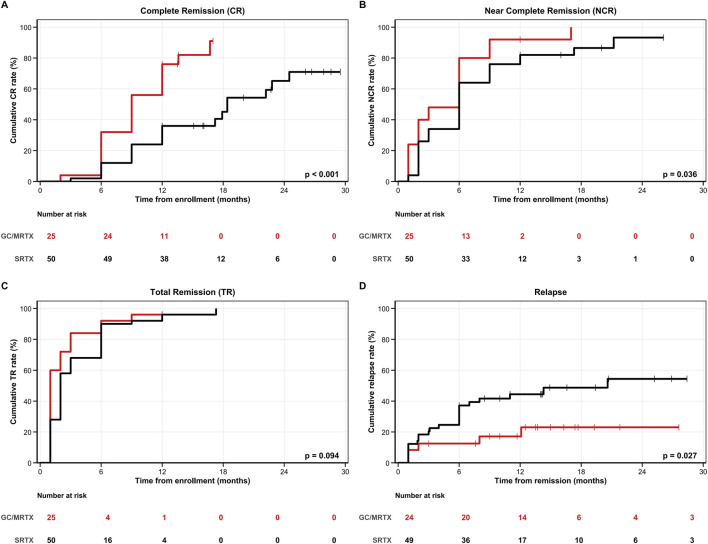
Kaplan–Meier analyses of clinical outcomes in the overall PMN cohort. **(A)** Complete remission (CR): The GC/MRTX group achieved significantly higher cumulative CR than the SRTX group (log-rank p < 0.001). **(B)** Near-complete remission (NCR): GC/MRTX showed superior cumulative NCR (log-rank p = 0.036). **(C)** Total remission (TR): Both groups had high remission rates, with a trend favoring GC/MRTX (p = 0.094). **(D)** Relapse-free survival: The GC/MRTX group demonstrated significantly lower cumulative relapse (p = 0.027). Numbers at risk are shown below each plot.

At 12 months, CR was achieved in 64.0% (16/25) of patients receiving GC/MRTX versus 22.0% (11/50) of those receiving SRTX (absolute risk difference, 0.42; 95% CI, 0.19 to 0.61; OR, 6.29; 95% CI, 2.15 to 18.40; p < 0.001) (Table 2). Median time to CR was significantly shorter in the GC/MRTX group (9.0 months [95% CI: 9.0–12.0] vs. 18.4 months [95% CI: 17.2–not reached], p < 0.001) than in the SRTX group. Kaplan–Meier analysis demonstrated higher cumulative CR in the GC/MRTX group (log-rank p < 0.001) (Figure 3A). NCR at 12 months was 88.0% (22/25) in the GC/MRTX group versus 70.0% (35/50) in the SRTX group (absolute risk difference, 0.18; 95% CI, −0.02 to 0.37; OR, 3.14; 95% CI, 0.85 to 11.59; p = 0.085). Cumulative NCR favored the MRTX group (log-rank p = 0.036) (Figure 3B). TR at 12 months reached 96.0% (24/25) versus 84.0% (42/50) (p = 0.258), with cumulative TR showing a trend toward GC/MRTX (log-rank p = 0.094) (Figure 3C). Relapse occurred in 0% (0/25) of patients receiving GC/MRTX versus 18.4% (9/49) of those receiving SRTX at the end of follow-up (p = 0.026). Kaplan–Meier analysis confirmed lower cumulative relapse risk in the GC/MRTX group (log-rank p = 0.027) (Figure 3D). Multivariable Cox regression identified GC/MRTX treatment as the strongest independent predictor of CR (adjusted HR 3.451, 95% CI 1.778–6.697, p < 0.001) after adjusting for baseline proteinuria (HR 0.917, 95% CI 0.854–0.984, p = 0.017) and other clinical parameters (Table 3).

**TABLE 2:** Comparison of primary and secondary clinical endpoints between the two treatment regimens.

Variables	GC/MRTX (n = 25)	SRTX (n = 50)	P Value
Follow-up duration			
Median follow-up (months)	17.0 (12.5, 25.6)	14.8 (12.0, 27.1)	0.310
Range	12.0–39.7	12.0–42.9	NA
Complete remission			
6 months, n (%)	7 (28.0)	6 (12.0)	0.161
12 months, n (%)	16 (64.0)	11 (22.0)	<0.001*
End of follow-up, n (%)	11 (44.0)	9 (18.0)	0.026*
Median time to CR	9.0 (9.0–12.0)	18.4 (17.2-NA)	<0.001*
Near complete remission			
6 months, n (%)	19 (76.0)	26 (52.0)	0.046*
12 months, n (%)	22 (88.0)	35 (70.0)	0.085
End of follow-up, n (%)	23 (92.0)	31 (62.0)	0.006*
Median time to NCR	6.0 (2.0–6.0)	6.0 (6.0–9.0)	0.036*
Total remission			
6 months, n (%)	23 (92.0)	41 (82.0)	0.419
12 months, n (%)	24 (96.0)	42 (84.0)	0.258
End of follow-up, n (%)	24 (96.0)	40 (80.0)	0.088
Median time to TR	1.0 (1.0–3.0)	2.0 (2.0–3.0)	0.094
Relapse rate			
End of follow-up, n (%)	0 (0)	9 (18.4)	0.026
Median time to relapse	NA	20.6 (7.0-NA)	0.027*

Data are presented as n (%) or median (IQR), unless otherwise specified. Time-to-event outcomes are presented as Kaplan–Meier median (95% CI) in months. Remission criteria: Complete remission (CR): 24-h urinary protein < 0.3 g/24 h, and eGFR decrease <25% from baseline; Partial remission (PR): proteinuria reduction ≥50% from baseline with 24-hour urinary protein <3.5 g, improvement or normalization of serum albumin, and stable kidney function; Near complete remission (NCR): proteinuria reduction >80% from baseline; Total remission (TR): achievement of CR or PR. Relapse: recurrence of nephrotic-range proteinuria (>3.5 g/24h) after achieving remission. Relapse rates are calculated based on patients who achieved remission. Time to event is presented as median (95% confidence interval) in months; NA indicates median not reached during follow-up. GC/MRTX: exposure-optimized strategy consisting of modified-interval rituximab plus structured glucocorticoid tapering and subsequent TDM-guided redosing; SRTX: standard rituximab monotherapy regimen (RTX 375 mg/m^2^ weekly ×4). *P < 0.05 indicates statistically significant differences between groups. Bold values indicate statistical significance (P < 0.05).

**TABLE 3:** Cox regression analysis for time to complete remission.

Variables	Univariable analysis		Multivariable analysis	
	HR (95% CI)	P Value	HR (95% CI)	P Value
Treatment regimen (GC/MRTX vs. SRTX)	3.514 (1.859–6.64)	<0.001*	3.451 (1.778–6.697)	<0.001*
Age (years)	0.995 (0.976–1.013)	0.566		
Sex (female vs. male)	1.301 (0.715–2.369)	0.389		
Body mass index (kg/m^2^)	1.012 (0.926–1.106)	0.791		
Baseline proteinuria (g/24 h)	0.936 (0.878–0.998)	0.043*	0.917 (0.854–0.984)	0.017*
Baseline albumin (g/L)	0.987 (0.932–1.044)	0.640		
Baseline creatinine (μmol/L)	0.991 (0.981–1.001)	**0.072**		
eGFR (mL/min/1.73 m^2^)	0.999 (0.99–1.009)	0.902		
Triglycerides (mmol/L)	1.11 (0.953–1.285)	0.183		
Total cholesterol (mmol/L)	1.03 (0.906–1.172)	0.650		
CD3^+^ T cell (cell/μL)	1 (1–1.001)	0.222		
CD4^+^ T cell (cell/μL)	1.001 (1–1.001)	0.046*	1.000 (0.999–1.001)	0.689
CD8^+^ T cell (cell/μL)	1 (0.998–1.001)	0.788		
CD19^+^ B cell (cell/μL)	1.001 (1–1.003)	0.016*	1.001 (0.999–1.003)	0.293
Anti-PLA2R antibody	0.834 (0.438–1.589)	0.581		
Hypertension	0.629 (0.352–1.125)	0.118		
Diabetes mellitus	0.896 (0.461–1.745)	0.747		
CardiovascularDisease	0.789 (0.789–8.425)	0.117		
CerebrovascularDisease	0.305 (0.073–1.27)	0.103		

Data are presented as hazard ratio (HR) with 95% confidence interval (CI). Multivariable model adjusted for treatment regimen, age, sex, body mass index, baseline proteinuria, baseline albumin, baseline creatinine, total protein, triglycerides, total cholesterol, HDL cholesterol, LDL cholesterol, and anti-PLA2R antibody status. Model performance: C-index = 0.730; AIC = 295.10; BIC = 317.69. *P < 0.05. Abbreviations: GC/MRTX, exposure-optimized strategy consisting of modified-interval rituximab plus structured glucocorticoid tapering and subsequent TDM-guided redosing; SRTX, standard rituximab regimen; eGFR, estimated glomerular filtration rate; HDL, high-density lipoprotein; LDL, low-density lipoprotein; PLA2R, phospholipase A2 receptor. Bold values indicate statistical significance (P < 0.05).

### 3.3 Longitudinal changes and immunological response

**FIGURE 4:**
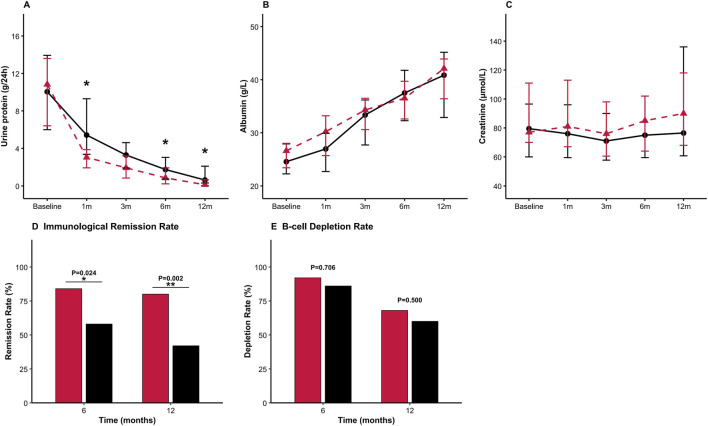
Longitudinal changes in clinical and immunologic indicators. **(A)** 24-hour urinary protein: Faster and deeper reduction with GC/MRTX at months 1 and 6 (*p < 0.05). **(B)** Serum albumin: Gradual improvement in both groups. **(C)** Serum creatinine: Stable kidney function throughout follow-up. **(D)** immunological remission < 14 RU/mL; complete immunological remission < 2 RU/mL: Higher rates in GC/MRTX at 6 and 12 months (p = 0.024, p = 0.002). **(E)** B-cell depletion (CD19^+^ <5 cells/μL): Similar depletion rates at 6 and 12 months.

Proteinuria declined faster in the GC/MRTX group throughout the follow-up, with significantly lower levels observed at 1, 6, and 12 months compared with those in the SRTX group (all p < 0.05) (Figure 4A). Serum albumin recovered more rapidly in the GC/MRTX group during the first 3 months, with both groups achieving similar levels by 12 months (Figure 4B). Creatinine levels remained stable throughout the follow-up in both groups (Figure 4C).

Complete Immunological remission (<2 RU/mL) at 6 months was 84% in the GC/MRTX group and 58% in the SRTX group (p = 0.024), evolving to 80% versus 42%, respectively, at 12 months (p = 0.002) (Figure 4D). B-cell depletion rates at 6 months were similar (92% vs. 82%, p = 0.706), declining to 68% versus 60%, respectively, at 12 months (p = 0.500) (Figure 4E).

**FIGURE 5:**
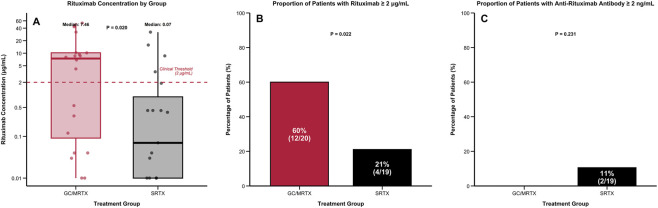
Serum rituximab exposure and anti-rituximab antibody detection at 6 months. **(A)** Serum RTX concentrations: Higher drug exposure in the GC/MRTX group (median 7.46 μg/mL vs. 0.07 μg/mL; p = 0.020). **(B)** Proportion with therapeutic exposure (≥2 μg/mL): 60% vs. 21% (p = 0.022). **(C)** Anti-RTX antibodies (ARAs): Detected only in SRTX patients (11%).

At 6 months, median serum rituximab concentration was 7.46 μg/mL (IQR: 0.94–11.77) in the GC/MRTX group versus 0.07 μg/mL (IQR: 0.03–1.07) in the SRTX group (p = 0.020) (Figure 5). Therapeutic concentrations (≥2 μg/mL) were maintained in 60.0% (15/25) versus 21.1% (10/50) of patients receiving GC/MRTX and SRTX, respectively (p = 0.022). In the GC/MRTX group, rituximab trough concentrations were assessed at Months 9 and 12 (Supplementary Figures 3A,B). The median trough RTX concentration was 4.21 μg/mL (IQR 1.87–6.56; n = 23) at Month 9 and 4.12 μg/mL (IQR 2.34–5.12; n = 23) at Month 12. The proportions of patients with trough RTX ≥2 μg/mL were 74% (17/23) and 83% (19/23), respectively. Additional RTX administration during follow-up is summarized in Supplementary Figure 2. In the GC/MRTX group, additional dosing occurred in 0/25 patients at month 6, 6/23 at month 9, and 5/23 at month 12 (n/N; denominators indicate evaluable patients at each time point) (Supplementary Figure 2A). In the SRTX group, retreatment was assessed at month 6, with 17/50 patients receiving retreatment (RTX 375 mg/m^2^ per infusion); no protocol-scheduled retreatment was planned or assessed at months 9 and 12, which is indicated as “–†” (Supplementary Figure 2A). Among SRTX patients who underwent month-6 retreatment (n = 17), all responders (PR, n = 8) received a single-infusion retreatment (8/8), whereas non-responders (NR, n = 9) received either a single infusion (6/9) or weekly ×2 retreatment (3/9), with weekly ×2 retreatment occurring exclusively in non-responders (Supplementary Figure 2B). ARAs were detected only in SRTX patients (11% vs. 0%, p = 0.231).

### 3.4 Comparison of efficacy in refractory PMN (RMN) subgroup

**FIGURE 6:**
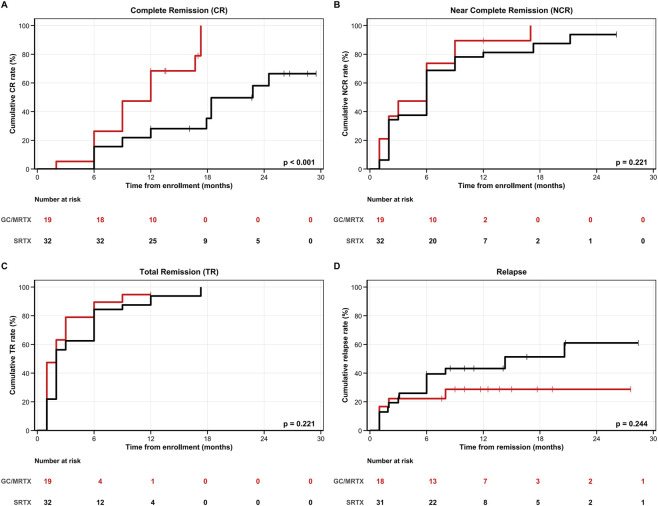
Kaplan–Meier analyses of clinical outcomes in the RMN subgroup. **(A)** Complete remission (CR): The GC/MRTX group achieved significantly higher cumulative CR (log-rank p < 0.001). **(B)** Near-complete remission (NCR): Higher in GC/MRTX but not statistically significant (log-rank p = 0.221). **(C)** Total remission (TR): No significant difference between groups. **(D)** Relapse-free survival: Lower relapse tendency in GC/MRTX (log-rank p = 0.244). Numbers at risk are shown below each plot.

Baseline characteristics were balanced among the 51 patients with refractory MN (GC/MRTX, n = 19; SRTX, n = 32) (Table 4). At 12 months, the GC/MRTX group achieved higher CR (52.63% vs. 18.75%; absolute risk difference, 0.34; 95% CI, 0.08 to 0.57; OR, 4.87; 95% CI, 1.40 to 16.90; p = 0.012) (Table 5). Median time to CR was 12.0 months (95% CI: 9.0–not reached) and 22.8 months (95% CI: 17.9–not reached) (p < 0.001) in the GC/MRTX and SRTX groups, respectively. Kaplan–Meier analysis demonstrated superior cumulative CR in the GC/MRTX group (log-rank p < 0.001) (Figure 6A).

**TABLE 4:** Baseline clinical characteristics of RMN.

Variables	GC/MRTX (n = 19)	SRTX (n = 32)	P Value
Demographic			
Age (y)	57.0 (49.5, 66.0)	55.5 (48.3, 65.5)	0.800
Male (%)	16 (84.21)	24 (75.00)	0.674
BMI (kg/m^2^)	24.2 (22.4, 26.6)	24.1 (22.2, 26.6)	0.812
Medical history			
Hypertension (%)	13 (68.42)	20 (62.50)	0.669
Diabetes mellitus (%)	4 (21.05)	4 (12.50)	0.679
Cardiovascular disease (%)	0 (0.00)	1 (3.12)	1.000
Cerebrovascular disease (%)	1 (5.26)	7 (21.88)	0.238
Clinical variables			
Urine protein (g/24 h)	12.12 (7.71, 13.68)	11.37 (9.63, 14.58)	0.650
Albumin (g/L)	26.70 (22.80, 27.85)	23.30 (21.12, 26.50)	0.144
Scr (μmol/L)	93.00 (75.00, 115.50)	84.50 (69.75, 97.25)	0.170
eGFR (mL/min/1.73 m^2^)	72.38 (51.03, 92.44)	88.65 (72.41, 99.95)	0.094
CD3^+^ T cell (cell/μL)	918.75 (695.54, 1,414.00)	1,349.24 (893.02, 1,563.72)	0.180
CD4^+^ T cell (cell/μL)	610.66 (454.50, 841.27)	814.06 (580.65, 1,033.03)	0.104
CD8^+^ T cell (cell/μL)	323.16 (194.06, 665.90)	462.07 (331.50, 557.66)	0.324
CD19^+^ B cell (cell/μL)	263.76 (155.99, 337.15)	213.91 (136.13, 316.17)	0.661
PLA2R ab* (RU/mL)	30.48 (10.79, 73.20)	61.72 (19.03, 128.23)	0.188
PLA2R ab positive (%)	12 (66.67)	24 (75.00)	0.529

Refractory membranous nephropathy (RMN) was defined as treatment-resistant and/or relapsing PMN: failure to achieve at least partial remission after an adequate prior course of immunosuppressive therapy, and/or relapse after a documented remission.

**TABLE 5:** Comparison of primary and secondary clinical endpoints between the two treatment regimens in RMN.

Variables	GC/MRTX	SRTX	P Value
Follow-up duration			
Median follow-up (months)	16.8 (12.00, 22.85)	12.00 (12.00, 27.17)	0.632
Complete remission			
6 months, n (%)	4 (21.05)	5 (15.62)	0.911
12 months, n (%)	10 (52.63)	6 (18.75)	0.012*
End of follow-up, n (%)	7 (36.8)	4 (12.5)	0.075
Median time to CR	12.0 (9.0-NA)	22.8 (17.9-NA)	<0.001*
Near complete remission			
6 months, n (%)	14 (73.68)	19 (59.38)	0.301
12 months, n (%)	16 (84.21)	23 (71.88)	0.508
End of follow-up, n (%)	18 (94.7)	21 (65.6)	0.020*
Median time to NCR	6.0 (2.0–9.0)	6.0 (3.0–9.0)	**0.221**
Total remission			
6 months, n (%)	17 (89.47)	24 (75.00)	0.371
12 months, n (%)	18 (94.74)	26 (81.25)	0.351
End of follow-up, n (%)	18 (94.7)	25 (78.1)	0.231
Median time to TR	2.0 (1.0–6.0)	3.0 (2.0–6.0)	0.221
Relapse rate			
End of follow-up, n (%)	0 (0)	6 (19.4)	0.073
Median time to relapse	NA	14.3 (6.0-NA)	0.244

This table presents a subgroup analysis of patients with refractory membranous nephropathy (RMN), defined as treatment-resistant and/or relapsing PMN: failure to achieve at least partial remission after an adequate prior course of immunosuppressive therapy, and/or relapse after a documented remission.

NCR in the GC/MRTX and SRTX groups at 12 months was 84.21% versus 71.88% (p = 0.508), respectively, reaching 94.7% versus 65.6% at the end of follow-up (p = 0.020) (Figure 6B). Total remission (TR) over follow-up is shown in Figure 6C. Relapse rates were numerically lower in the GC/MRTX group (0% vs. 19.4%, p = 0.073), although the difference was not statistically significant (log-rank p = 0.244) (Figure 6D).

**FIGURE 7:**
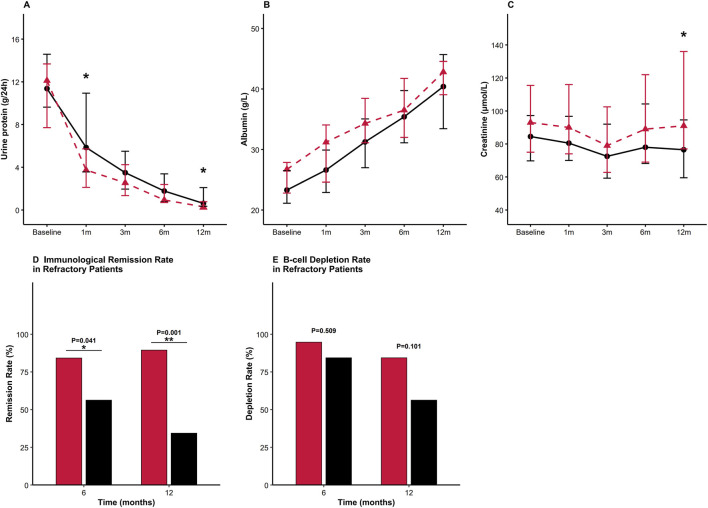
Longitudinal clinical and immunologic responses in RMN. **(A)** Proteinuria: Faster reduction with GC/MRTX at months 1 and 12 (*p < 0.05). **(B)** Serum albumin: Comparable improvement in both groups. **(C)** Serum creatinine: Stable overall; significant difference at month 12 (*p < 0.05). **(D)** Immunologic remission: GC/MRTX markedly superior at 6 and 12 months (p = 0.041 and p < 0.001). **(E)** B-cell depletion: Similar between groups.

In the refractory subgroup, the reduction in proteinuria mirrored the pattern observed in the overall cohort, with a steeper decline evident at 1 month in the GC/MRTX group (4 g/24 h vs. 7 g/24 h, p < 0.05) (Figure 7A). Serum albumin improved over time in both groups (Figure 7B), and serum creatinine remained overall stable, with a between-group difference observed at month 12 (Figure 7C). Immunological remission observed in the GC/MRTX group at 12 months was markedly superior (89.47% vs. 34.38%, p = 0.001) (Figure 7D) to that in the SRTX group. B-cell depletion rates were comparable between the two groups (94.12% vs. 84.38% at 6 months, p = 0.609) (Figure 7E).

### 3.5 Safety and tolerability

Both treatment regimens were well tolerated. The overall incidence of adverse events was similar between the GC/MRTX group (20% [5/25]) and the SRTX group (22% [11/50], p = 0.792) (Table 6). The most common events were infections (20% vs. 12%, p = 0.479), including respiratory tract infections (12% vs. 6%, p = 0.665) and urinary tract infections (8% vs. 6%, p = 1.000). All infections were mild and resolved with oral antibiotics. Leukopenia was rare, occurring in 0/25 (0%) patients in the GC/MRTX group and 1/50 (2.0%) in the SRTX group (p = 1.000). No serious adverse events occurred in either group.

**TABLE 6:** Summary of adverse events during treatment in both groups.

Variables(N)	GC/MRTX (n = 25)	SRTX (n = 50)	P Value
Total AEs (%)	5 (20.0)	11 (22.0)	0.792
Infections (%)	5 (20.0)	6 (12.0)	0.479
Respiratory tract infection (%)	3 (12.0)	3 (6.0)	0.665
Urinary tract infection (%)	2 (8.0)	3 (6.0)	1.000
Increase of liver enzymes (%)	0	1 (2.0)	1.000
Leukopenia	0 (0.0)	1 (2.0)	1.000
Diabetes	0 (0.0)	2 (4.0)	0.544
Cardiovascular events	0 (0.0)	1 (2.0)	1.000

Data are presented as n (%). Repeated occurrences of the same adverse event in a single patient were counted only once. Comparisons between groups were performed using Fisher’s exact test. AE: adverse event.

## 4 Discussion

In this propensity score–matched multicenter retrospective study, we evaluated an exposure-optimized strategy comprising modified-interval RTX, a structured glucocorticoid taper, and subsequent TDM-guided redosing. This strategy was associated with higher and earlier complete remission, deeper immunologic responses, and lower relapse compared with the standard RTX regimen without concomitant glucocorticoids, with a comparable safety profile. Because multiple components were implemented as a package, the observed benefit should be interpreted at the strategy level, rather than attributed to the initial RTX schedule alone. Conceptually, the strategy was designed to mitigate the early 2–3 months pharmacokinetic vulnerability window by reinforcing and maintaining effective RTX exposure.

RTX has become a central therapy for PMN, yet remission rates across clinical trials and real-world studies remain inconsistent. Even among landmark randomized trials, CR rates varied substantially, ranging from approximately 16% in RI-CYCLO to nearly 60% in MENTOR (Fervenza et al., 2019; Scolari et al., 2021), despite similar diagnostic criteria adopted by the two trials. Our SRTX cohort demonstrated a 12-month CR rate of only 22%, closely mirroring the modest efficacy reported in several real-world studies (Gao et al., 2021; Zhang et al., 2023; Xu et al., 2025) and reinforcing that fixed oncologic dosing may not adequately address the distinctive immunologic and pharmacokinetic context of PMN.

Mechanistic insights increasingly suggest that suboptimal drug exposure may be an important contributor. Accelerated RTX clearance driven by urinary immunoglobulin loss, altered IgG metabolism, and heightened immunologic activity frequently leads to subtherapeutic concentrations within two to three months after infusion (Dahan et al., 2017; Fogueri et al., 2019; Boyer-Suavet et al., 2019; Hartinger et al., 2024). Prospective pharmacokinetic analyses consistently show early B-cell reconstitution and rebounds in anti-PLA2R titers as RTX levels decline (Dahan et al., 2017; Beck et al., 2011; Ruggenenti et al., 2015). Our multicenter pharmacokinetic monitoring corroborates these observations: more than 70% of patients exhibited insufficient RTX exposure, and 25% developed ARAs, with serum levels dropping precisely during the period when immunologic rebound is most likely. These findings highlight a structural vulnerability in SRTX regimens and provide plausible mechanistic context for therapeutic inconsistency (Seitz-Polski et al., 2019; Teisseyre et al., 2021). Real-world evidence from Chinese centers further reflects this heterogeneity, with remission outcomes differing substantially despite similar patient characteristics. Variability across centers was closely linked to differences in dosing intensity and baseline immunologic burden (Gao et al., 2021; Zhang et al., 2023; Xu et al., 2025), supporting the concept that inconsistent RTX exposure, rather than intrinsic drug resistance, largely determines treatment outcome. Collectively, these observations underscore the need for exposure-optimized RTX strategies that align more closely with the pharmacokinetic behavior of the drug in PMN.

The GC/MRTX regimen was therefore designed to reinforce RTX exposure during the early pharmacokinetic vulnerability window through an intensified initial course (days 1, 15, 30, and 120) followed by pharmacokinetic-guided maintenance dosing based on serum RTX monitoring every 3 months, with additional infusions administered when concentrations fell below 2 μg/mL. This exposure-optimized approach was integrated with short-term glucocorticoid tapering to enhance early disease control (Li et al., 2024). In this multicenter propensity-matched cohort, GC/MRTX achieved substantially higher and earlier remission than SRTX. At 12 months, CR was 64% with GC/MRTX—nearly threefold higher than that achieved with SRTX and exceeding the CR rates reported in most major RTX trials (Fervenza et al., 2019; Scolari et al., 2021; Fernández-Juárez et al., 2021). Immunologic remission occurred earlier and more consistently with GC/MRTX, demonstrating that maintaining adequate RTX exposure is consistent with improved suppression of anti-PLA2R antibodies and durable immunologic control. Peripheral B-cell depletion assessed by a dichotomized CD19^+^ threshold (<5 cells/μL) was similar between groups at months 6 and 12. However, this binary measure at scheduled visits does not capture depletion depth or time-to-reconstitution. Therefore, we interpret these depletion data descriptively and avoid mechanistic attribution based on peripheral depletion status. Importantly, the advantages of GC/MRTX extended beyond the 12-month framework. During longer follow-up, CR and near-complete remission remained higher in the GC/MRTX group, and relapse occurred only in SRTX recipients. Although follow-up intervals varied in real-world practice, the persistence of benefit across both clinical and immunologic domains supports the durability of the GC/MRTX strategy. Early reductions in proteinuria and improvements in serum albumin were also more pronounced, consistent with the hypothesis that short-term glucocorticoids may stabilize glomerular permeability during the lag period before RTX exerts full immunologic effects (Li et al., 2024).

The benefits of GC/MRTX were particularly evident in patients with refractory disease. Previous studies reported limited efficacy of RTX in this population, often with CR rates below 20%–30% (Gao et al., 2021; Zhang et al., 2023; Xu et al., 2025). In contrast, GC/MRTX achieved a 12-month CR rate of 52.6% in refractory patients, substantially outperforming both SRTX (18.8%) and most historical RTX regimens (Gao et al., 2021; Zhang et al., 2023; Xu et al., 2025). These findings support an emerging paradigm: many cases labeled “refractory” may reflect inadequate RTX exposure rather than true immunologic nonresponsiveness, however, this interpretation remains hypothesis-generating in this retrospective study with a multi-component intervention. This is also consistent with reports of robust responses to more potent anti-CD20 agents such as obinutuzumab in RTX-poor responders (Su et al., 2025).

This study has limitations. Residual confounding cannot be fully excluded given the retrospective design despite propensity score matching. B-cell monitoring was limited to a dichotomized peripheral CD19^+^ depletion status at scheduled visits, precluding assessment of depletion depth, duration, and time-to-reconstitution. Heterogeneity may have arisen from variability in visit timing, monitoring frequency, treatment adherence, and incomplete capture of long-term follow-up across centers. Retreatment/redosing patterns also differed between groups, introducing potential confounding by treatment intensity and indication. Although GC/MRTX was “structured” as a prespecified strategy framework (planned RTX time points, prednisone taper milestones, and TDM-guided redosing rules), real-world implementation was imperfect, with common timing deviations (predominantly delays), particularly for later infusions, which may have altered intended exposure coverage and affected PK, immune control, and outcomes. These impacts cannot be quantified retrospectively; thus, findings are strategy-level associations requiring prospective RCT validation.

Building upon these promising findings, we have already initiated a prospective, multicenter randomized controlled trial across the province to prospectively evaluate these results and evaluate long-term outcomes. Our work ultimately aims to establish a new, more effective treatment paradigm to improve clinical management for patients with PMN.

## 5 Conclusion

In this propensity score–matched multicenter retrospective cohort, an exposure-optimized strategy combining interval RTX dosing, structured glucocorticoid tapering, and TDM-guided redosing was associated with higher and faster complete remission, deeper immunologic response, and lower relapse in PMN, including refractory cases. These findings support further prospective evaluation to confirm efficacy and clarify the independent contributions of strategy components before changes in practice are recommended.

## Data Availability

The original contributions presented in the study are included in the article/Supplementary Material, further inquiries can be directed to the corresponding authors.
