# Supplementary material for: Optimized RTX strategy plus structured glucocorticoid tapering for primary membranous nephropathy: a multicenter propensity score-matched cohort study
Source: Front Mol Biosci. 2026 Mar 4;13:1770916. doi: 10.3389/fmolb.2026.1770916 (PMC12996836; doi:10.3389/fmolb.2026.1770916)
Supplement: Supplementary file 1 [file Supplementaryfile1.docx]

**Supplementary Table S1A.** **Timing deviations between planned and actual rituximab administrations (GC/MRTX vs SRTX)**

| \| **Regimen** \| \| --- \| | Planned timepoint | N (administrations) | | deviation, n (%) | Earlier (negative), n (%) |  | Delayed (positive), n (%) | Deviation (days), median (IQR) |
| --- | --- | --- | --- | --- | --- | --- | --- | --- | --- |
| GC/MRTX | Infusion 1 | | 25 | 0 (0) | 0 (0) |  | 0 (0) | 0 (0–0) |
| GC/MRTX | Infusion 2 | | 25 | 16 (64) | 2 (8) |  | 14 (56) | 1 (0–2) |
| GC/MRTX | Infusion 3 | | 25 | 19 (76) | 0 (0) |  | 19 (76) | 2 (1–2) |
| GC/MRTX | Infusion 4 | | 25 | 23 (92) | 4 (16) |  | 19 (76) | 6 (2–8) |
| SRTX | Week 1 | | 50 | 0 (0) | 0 (0) |  | 0 (0) | 0 (0–0) |
| SRTX | Week 2 | | 50 | 20 (40) | 0 (0) |  | 20 (40)* | 0 (0–1) |
| SRTX | Week 3 | | 50 | 29 (58) | 0 (0) |  | 29 (58)* | 1 (0–1) |
| SRTX | Week 4 | | 50 | 29 (58) | 0 (0) |  | 29 (58)* | 1 (0–2) |

Note:Timing deviations were assessed for the fixed induction schedule only (GC/MRTX: days 1/15/30/120; SRTX: weekly ×4). TDM-guided redosing infusions were not included because they were triggered by serum RTX concentrations rather than prespecified calendar dates.*In the SRTX cohort, **all non-zero deviations were delays** (no earlier-than-planned administrations were observed).

**Table S1B. Patient-level summary of timing deviations**

| \| Regimen \| \| --- \| | N (patients) | ≥1 deviation, n (%) | | ≥1 delay, n (%) | ≥1 earlier, n (%) |  | Both earlier and delayed across visits, n (%) | Patient-level maximum absolute deviation (days) |
| --- | --- | --- | --- | --- | --- | --- | --- | --- | --- |
| GC/MRTX | 25 | | 23 (92) | 22 (88) | 6 (24) |  | 5 (20) | 6 (2–8) |
| SRTX | 50 | | 29 (58) | 29 (58) | 0 (0) |  | 0 (0) | 1 (0–2) |

**Abbreviations:** GC, glucocorticoids; RTX, rituximab; **GC/MRTX,** exposure-optimized strategy consisting of modified-interval rituximab plus structured glucocorticoid tapering and subsequent TDM-guided redosing (fixed induction schedule: days 1/15/30/120); **SRTX,** standard rituximab monotherapy regimen (weekly ×4); IQR, interquartile range; TDM, therapeutic drug monitoring.

**Supplementary Table S2. Covariate balance before and after propensity score matching (PSM).**

| \| Variable \| \| --- \| | Before PSM: SRTX (n=74) | Before PSM: GC/MRTX (n=30) | | SMD (Before) | After PSM: SRTX (n=50) |  | After PSM: GC/MRTX (n=25) | SMD (After) |
| --- | --- | --- | --- | --- | --- | --- | --- | --- | --- |
| Age, years, median | 56.50 (42.25, 68.50) | | 58.00 (50.00, 66.50) | 0.142 | 55.50 (46.25, 67.00) |  | 59.00 (50.00, 67.00) | 0.126 |
| 24‑h proteinuria, g/24 h | 9.27 (6.00, 13.82) | | 11.26 (6.60, 13.72) | 0.175 | 10.05 (6.00, 13.93) |  | 10.83 (6.41, 13.60) | 0.024 |
| Serum albumin, g/L | 23.90 (20.97, 27.90) | | 27.00 (23.42, 29.55) | 0.404 | 24.55 (22.25, 27.90) |  | 26.60 (23.40, 28.00) | 0.177 |
| Serum creatinine, μmol/L | 83.15 (60.00, 112.75) | | 76.50 (65.75, 101.00) | −0.533 | 79.50 (60.00, 96.50) |  | 77.00 (70.00, 111.00) | 0.154 |
| eGFR, mL/min/1.73 m² | 93.45 (63.24, 106.52) | | 87.92 (57.01, 102.47) | −0.202 | 90.62 (71.19, 102.61) |  | 86.40 (56.66, 102.61) | −0.198 |
| CD19+ B cells, cells/μL | 239.06 (169.25, 345.50) | | 274.10 (176.09, 329.35) | −0.106 | 248.75 (155.50, 327.11) |  | 275.42 (169.68, 331.58) | −0.028 |
| Sex, male,n (%) | 56 (75.68) | | 19 (63.33) |  | 35 (70.00) |  | 17 (68.00) | −0.043 |

**Note: Continuous variables are presented as median (IQR); categorical variables as n (%).SMD = standardized mean difference; smaller absolute values indicate better balance.Conventional thresholds: |SMD| <0.10 (excellent), 0.10–0.20 (acceptable), >0.20 (residual imbalance).**
