# Supplementary material for: Optimized RTX strategy plus structured glucocorticoid tapering for primary membranous nephropathy: a multicenter propensity score-matched cohort study
Source: Front Mol Biosci. 2026 Mar 4;13:1770916. doi: 10.3389/fmolb.2026.1770916 (PMC12996836; doi:10.3389/fmolb.2026.1770916)
Supplement: Supplementary file 2 [file Image2.pdf]

Supplementary Figure 2. Additional rituximab administration during follow-up

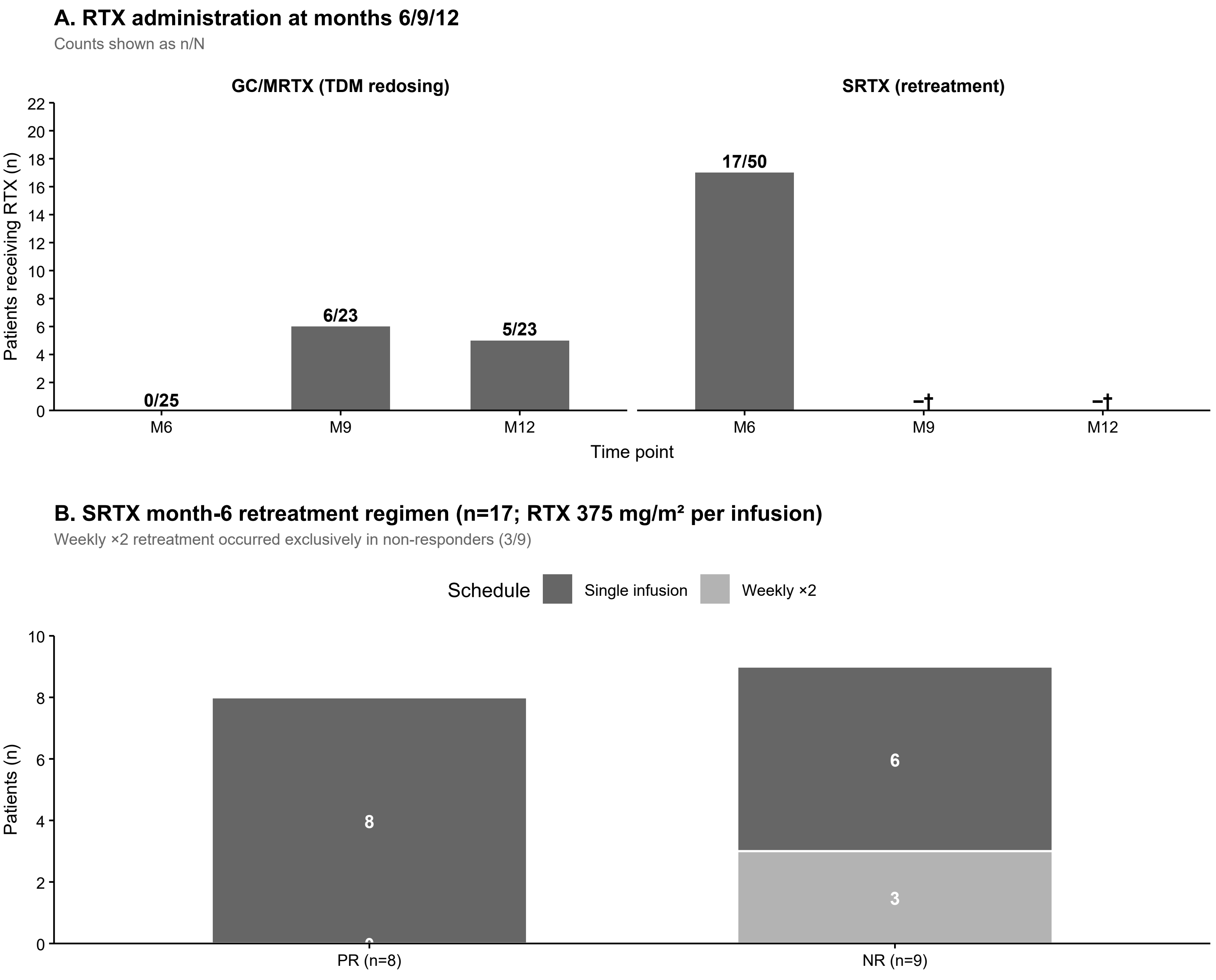

Definition of evaluable (N): N includes patients with available data at the specified visit; patients with missing visit data were excluded from N.

GC/MRTX: TDM-guided redosing was triggered by serum RTX <2 μg/mL and given as a single infusion (375 mg/m²).

†SRTX: no protocol-scheduled retreatment at months 9/12 (retreatment assessed at month 6).
