# Supplementary material for: Optimized RTX strategy plus structured glucocorticoid tapering for primary membranous nephropathy: a multicenter propensity score-matched cohort study
Source: Front Mol Biosci. 2026 Mar 4;13:1770916. doi: 10.3389/fmolb.2026.1770916 (PMC12996836; doi:10.3389/fmolb.2026.1770916)
Supplement: Supplementary file 3 [file Image3.pdf]

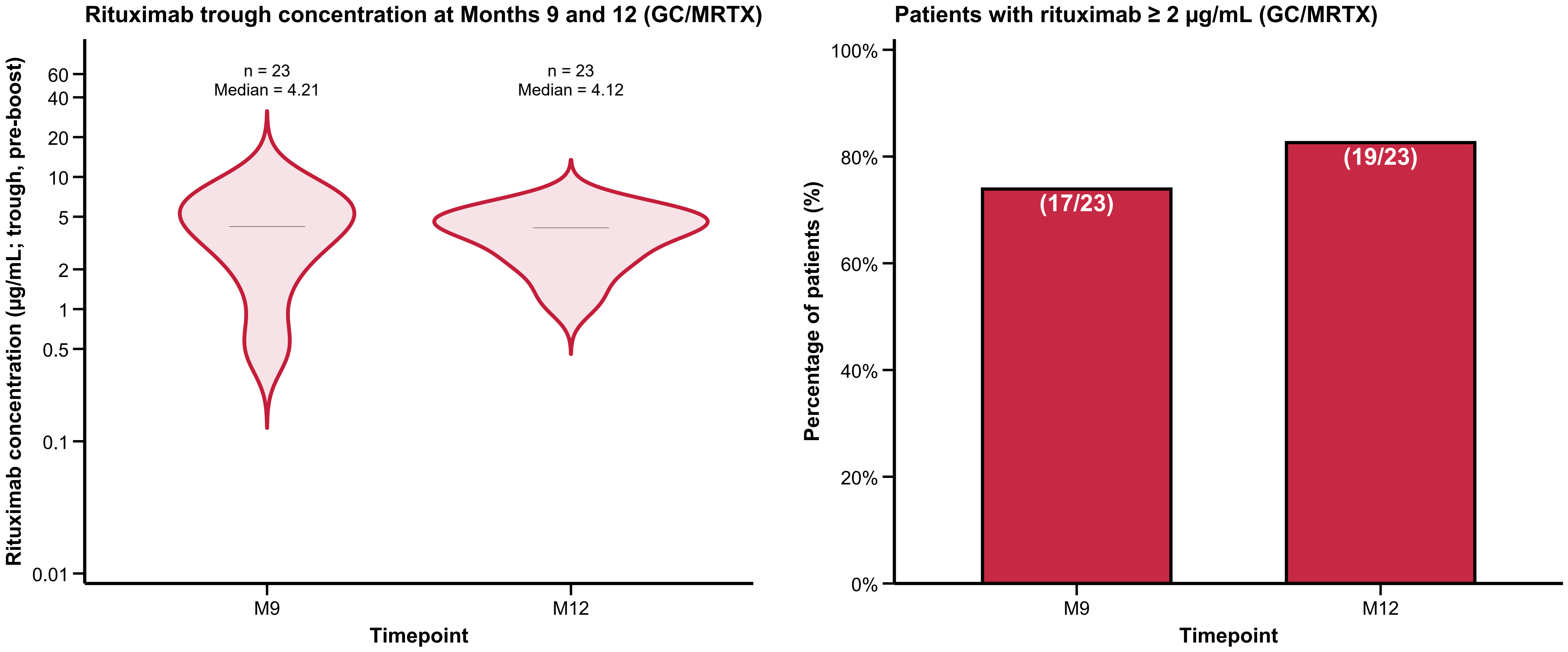

Supplementary Figure 3. Rituximab trough concentrations at Months 9 and 12 in the GC/MRTX group.
(A) Violin plots of trough concentrations (pre-boost); ticks indicate medians. Paired Wilcoxon signed-rank test (two-sided; n = 23), p = 0.659.
(B) Patients with trough concentration ≥ 2 µg/mL; denominators indicate patients with available measurements at each visit.
