# Supplementary material for: Optimized RTX strategy plus structured glucocorticoid tapering for primary membranous nephropathy: a multicenter propensity score-matched cohort study
Source: Front Mol Biosci. 2026 Mar 4;13:1770916. doi: 10.3389/fmolb.2026.1770916 (PMC12996836; doi:10.3389/fmolb.2026.1770916)
Supplement: Supplementary file 4 [file Image1.pdf]

Supplementary Figure 1. Real-World Rituximab TDM Data from China

Wuxi Zhengze Medical Laboratory, Jiangsu Province (n=429 dual-tested patients)

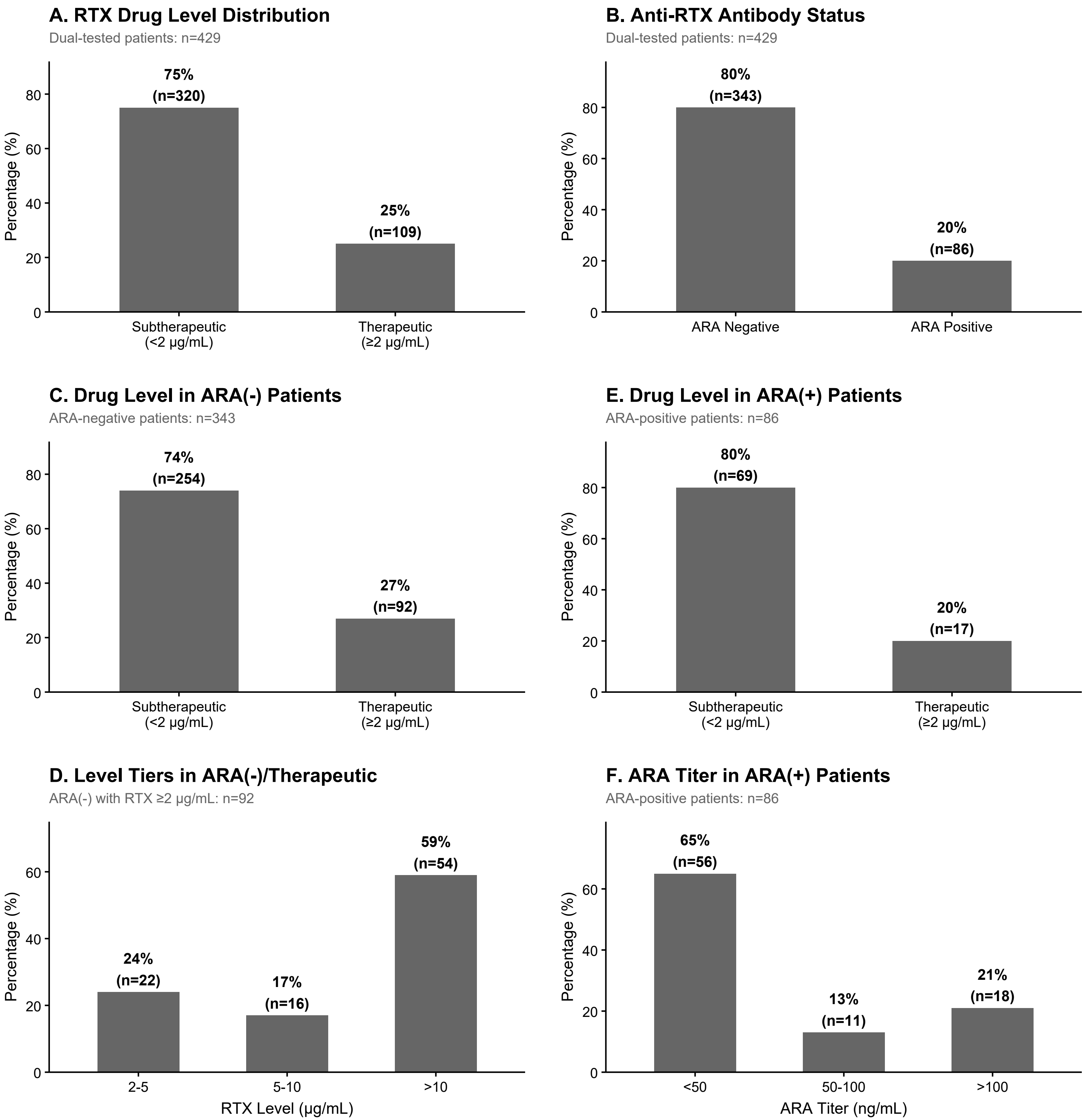

RTX: rituximab; ARA: anti-rituximab antibody. Therapeutic level: RTX ≥2 μg/mL.
